# Socially‐Based Emotion Dysregulation Among Hearing‐Impaired Adolescents: An Event‐Related Potential Study

**DOI:** 10.1002/brb3.71080

**Published:** 2025-11-21

**Authors:** Panpan Zhang, Huang Gu, Minghui Wang

**Affiliations:** ^1^ Department of Psychology Henan University Kaifeng China; ^2^ Department of Psychology South China Normal University Guangzhou China

**Keywords:** attention allocation, elaborated processing, event‐related potentials, socially‐based emotion regulation

## Abstract

**Purpose:**

Hearing impairment adversely affects the development and maturation of the ventrolateral prefrontal cortex, which is a region critical for social emotion regulation. The present study aimed to examine how hearing impairment influences socially‐based emotion regulation in adolescents or its underlying neural mechanisms.

**Method:**

To address this gap, the present study recorded event‐related potentials (ERP) while participants viewed images depicting social exclusion or used emotion regulation strategies to down‐regulate their emotional responses.

**Finding:**

Behavioral results indicated that normal‐hearing adolescents reported a significant reduction in negative affect when employing regulation strategies, an effect not observed in hearing‐impaired adolescents. ERP data indicated that compared to passive viewing, expressive suppression elicited reduced amplitudes in the central‐parietal LPP and P3 components among normal‐hearing adolescents. This modulation was attenuated in those with hearing impairment.

**Conclusion:**

These results suggest that deficits in both attention allocation and elaborative emotional processing may underlie the impaired down‐regulation of socially triggered negative emotions in adolescents with hearing loss.

## Graphical abstract

This graphical abstract illustrates the study “Neural Mechanisms Underlying Emotional Dysregulation in Hearing‐Impaired Adolescents”. We compared hearing‐impaired adolescents and normal‐hearing adolescents using an emotion regulation task while recording EEG. The results indicate that hearing impairment leads to deficits in attention allocation and elaborative emotional processing, ultimately contributing to emotion dysregulation.

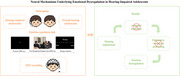

## 1 Introduction

According to the 2021 World Hearing Report, “disabling hearing loss” is defined herein: for adults, it refers to a pure‐tone hearing threshold of ≥ 40 decibels hearing level (dB HL) in the better ear; for children, it denotes a pure‐tone hearing threshold of ≥ 30 dB HL in the better ear. Based on this definition, over 430 million people worldwide experience disabling hearing loss, accounting for more than 5% of the global population. This number is projected to rise to 700 million by 2050. A growing body of evidence indicates that hearing loss can adversely affect language development, communication abilities, and social functioning (Cupples et al. 2018; Fitzpatrick et al. 2019; Socher et al. 2019; Walker et al. 2020). These challenges often contribute to difficulties in social interaction among the hearing‐impaired population (Olsson et al. 2021), which can lead to feelings of exclusion and hinder adaptation to social life.

Social exclusion occurs when an individual is ignored or rejected by a social group (Reinhard et al. 2020). This form of interpersonal rejection often elicits negative emotional experiences that share neural correlates with physical pain, including activation in the dorsal anterior cingulate cortex and the insula (Eisenberger 2012; Jauch et al. 2022; Zhao et al. 2021). Emotion regulation refers to the conscious and unconscious efforts by which individuals modulate their emotional experiences, including the selection, timing, and manifestation of their feelings (Gross 1998b), and has been shown to mitigate negative emotional experiences triggered by social exclusion scenarios (He et al. 2018, 2021; Zhao et al. 2021). Event‐related potentials (ERPs), with high temporal resolution, offer a means to objectively track the millisecond‐level dynamics of down‐regulating negative social emotion. In particular, the P3 and late positive potential (LPP) components are widely used in emotion regulation research. While the LPP is considered a sustained extension of the P3, both are modulated during the down‐regulation of negative social emotion, showing changes in amplitude, latency, and scalp distribution (He et al. 2020; Zhao et al. 2021). Critically, these components reflect distinct stages of emotional processing: the P3 is associated with attentional allocation to emotional stimuli, whereas the LPP reflects sustained elaborative processing (Hajcak et al. 2010).

Adolescence is a transitional period characterized by heightened sensitivity to social feedback and increased complexity in social environments. Compared to other age groups, adolescents are more vulnerable to emotional disorders such as depression and anxiety (Rapee et al. 2019). This vulnerability is especially pronounced among hearing‐impaired adolescents, who exhibit higher rates of emotional disorders relative to their hearing peers (Cejas et al. 2021). One potential explanation is impaired emotion regulation in response to unfavorable social exchanges. Supporting this, Wang et al. (2016) found that hearing loss disrupts the maturation of the ventrolateral prefrontal cortex (VLPFC), a region crucial for regulating negative social emotion (He et al. 2020; Li et al. 2022). Thus, dysregulated responses to negative social emotion may underlie the elevated risk of emotional disorders in hearing‐impaired adolescents. Yet, few studies have examined abnormalities in emotion regulation among hearing‐impaired adolescents, focusing on negative emotion induced by unfavorable social exchanges. Moreover, the neural mechanisms of this dysregulation remain poorly understood.

To address this gap, the present study drew on Gross's process model of emotion regulation, a foundational framework in the field. This model (Gross 1998a, 1998b) proposes that emotion regulation strategies operate at distinct temporal stages of emotional processing: cognitive reappraisal falls under the antecedent‐focused “cognitive change” strategy, while expressive suppression is categorized as the response‐focused “response modulation” strategy. Guided by this model, the study mainly examined two common emotion regulation strategies (cognitive reappraisal and expressive suppression) in hearing‐impaired adolescents who hold audiogram results issued by government‐recognized hospitals. Using ERPs, we aimed to characterize the neural signatures of downregulating negative social emotion in this population. We hypothesized that hearing‐impaired adolescents would exhibit impaired emotion regulation in response to negative social emotion, as reflected in reduced modulation of negativity ratings and ERP amplitudes (specifically P3 and LPP) compared to normal‐hearing adolescents.

## 2 Materials and Methods

### 2.1 Participants

The study included 35 hearing‐impaired adolescents (19 males) and 35 hearing controls (17 males), all between 11 and 16 years of age. All hearing‐impaired participants were recruited from a public special school, where they attend alongside students with other types of disabilities, though they are in separate classrooms. This special school accepts students who have audiogram results from government‐recognized hospitals. Both hearing‐impaired and hearing control participants were right‐handed, had normal or corrected‐to‐normal vision, and had no history of neurological or mental disorders, as confirmed by parental reports. Due to excessive head movement artifacts, 7 participants (2 hearing‐impaired and 5 hearing controls) were excluded from further analysis. As summarized in Table 1, the two groups did not differ significantly in gender or age (all *p*s > 0.05). Prior to the experiment, written informed consent was obtained from parents, and oral assent was provided by each adolescent. All participants received compensation upon completion of the experiment.

**TABLE 1:** Demographic characteristics of hearing‐impaired adolescents and hearing controls.

	Hearing‐impaired adolescents	Hearing controls	*t*/*χ* ^2^
Age (years)	13.76 (1.46)	13.40 (0.77)	1.23
Gender (male/female)	18/15	15/15	0.13
Handedness (right/left)	33/0	30/0	—
Communication mode	Sign language	Oral language	—

### 2.2 Experimental Materials and Procedure

The open‐access Image Database of Social Inclusion and Exclusion in Young Asian Adults (ISIEA; Zheng et al. 2022) provides suitable materials for socially oriented research. Previous studies on emotion regulation have used social exclusion images from ISIEA to elicit negative social emotion (He et al. 2020). However, since these earlier findings were based on college student samples, it remains unclear whether the ISIEA materials are equally appropriate for use with hearing‐impaired adolescents. To evaluate this, 15 social inclusion and 15 social exclusion images were randomly selected from the ISIEA database and rated by 45 adolescents who did not participate in the main experiment (17 hearing‐impaired and 28 normal‐hearing controls). Using a 9‐point scale ranging from 1 (most negative) to 9 (most positive), participants provided valence ratings. A paired‐samples *t*‐test revealed that both groups reported significantly more social negativity when viewing social exclusion images compared to social inclusion images (hearing‐impaired: social exclusion = 2.68 ± 1.21, social inclusion = 7.88 ± 1.48, *t*(16) = ‐9.56, *p* < 0.001; hearing controls: social exclusion = 2.75 ± 0.75, social inclusion = 7.09 ± 0.84, *t*(27) = ‐17.82, *p* < 0.001). These results support the validity of ISIEA for inducing negative social emotion in adolescent populations.

A total of 47 social exclusion images were randomly selected from the ISIEA database. Among these, two images were used in a practice phase, and the remaining 45 were assigned to the formal experiment. These 45 images were divided into three sets, each corresponding to one of the experimental conditions: passive viewing, cognitive reappraisal, and expressive suppression.

During the practice phase, two social exclusion images were presented. Participants were instructed to use either cognitive reappraisal or expressive suppression to downregulate their negative emotion while viewing each image. Specifically, in the reappraisal condition, they were asked to reinterpret the scene in a positive or neutral manner. In the suppression condition, they were told to inhibit their emotional expressions and conceal their feelings from external observers. Only after researchers confirm that participants have understood the instructions can they proceed to the formal experiment.

In the formal experiment, the 45 social exclusion images were divided into three sets, each aligned with one of the three conditions. The experiment consisted of three blocks, each corresponding to one condition, and each block contained 15 images, each of which was presented twice. To minimize potential carry‐over effects of emotion regulation, the passive viewing block was always administered first, consistent with prior studies (He et al. 2020; Zhao et al. 2021). The order of the reappraisal and suppression blocks was counterbalanced across participants.

**FIGURE 1:**
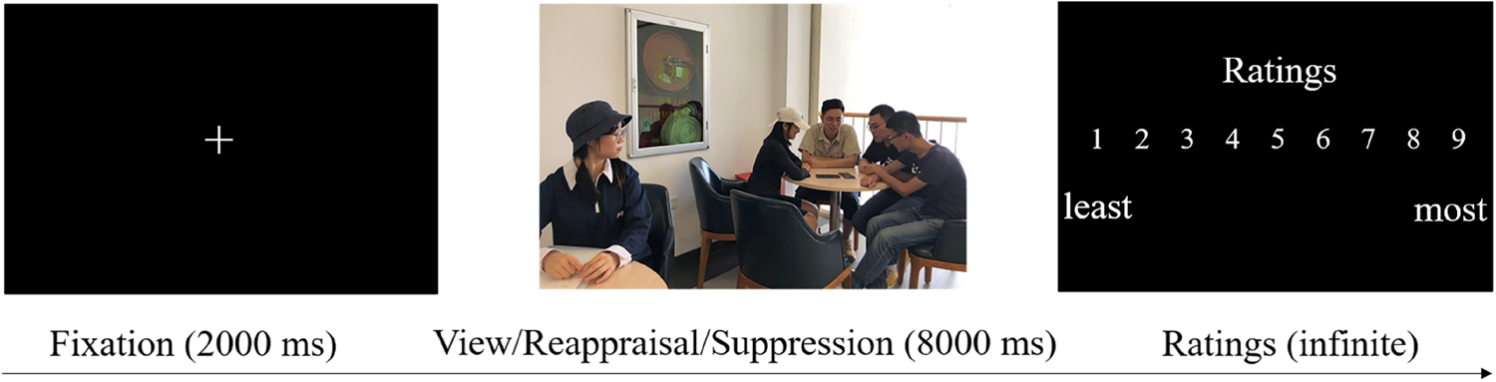
The experimental flow of a single trial in the emotion regulation task.

As illustrated in Figure 1, each trial began with a 2000 ms fixation cross, followed by an 8000 ms presentation of a social exclusion image. During image presentation, participants were instructed to either view the image passively or implement the specified emotion regulation strategy. The condition‐specific instructions were as follows: in the viewing condition, participants were asked to naturally experience any emotion elicited by the image; in the reappraisal condition, they reinterpreted the scene in a positive or neutral way; and in the suppression condition, they inhibited their emotional responses and concealed outward expressions. After the image disappeared, participants rated the intensity of their negative emotion on a 9‐point scale (1 = least negativity, 9 = most negativity) by selecting the corresponding number with the mouse.

### 2.3 EEG Recording and Analysis

EEG data were recorded using a 64‐channel amplifier (EGI, Eugene, OR, United States). The online reference electrode was located in the vertex channel. The EEG signals were digitized at a sampling rate of 1 kHz. The electrooculogram (EOG) was recorded by two vertical facial electrodes (below the left eye and the right eye) and two horizontal facial electrodes (the left side of the left eye and the right side of the right eye). All electrode impedances were maintained below 50 kΩ.

Offline EEG data were processed using MATLAB R2020b in the following steps. First, the offline data were re‐referenced to the linked mastoids, basspass filtered with a 0.1 to 30 Hz and filtered using a 50 Hz notch filter. The independent component analysis (ICA) was used to remove ocular artifacts. Continuous EEG data were epoch at a 200 ms pre‐stimulus interval voltage and a 1500 ms post‐stimulus interval. The baseline correction was performed based on 200 ms before the onset of stimuli. Epochs with artifacts exceeding ± 100 µv were excluded from further analyses. The results of repeated‐measures ANOVA showed that the number of remaining epochs in each condition was not different across the groups (*F*(2, 122) = 0.12, *p* > 0.05; see Supplementary File Table S1).

Based on grand‐mean ERP topographies and previous studies (Hajcak et al. 2010; Sun et al. 2020), the present study calculated the mean amplitudes for P3 (250‐350 ms) and LPP (400‐1500 ms) at central‐parietal areas (5 sites: CP1, CP2, P3, P4, and Pz).

### 2.4 Statistics

To examine the emotional regulation effect, a 2 (group: hearing‐impaired adolescents, hearing controls) by 3 (condition: view, reappraisal, suppression) repeated‐measures ANOVA was conducted using SPSS software ver. 21.0. For significance main effects and interactions, post‐hoc tests were performed using Bonferroni correction. The significant level was set at *p* < 0.05.

## 3 Results

### 3.1 Behavioral Results

**FIGURE 2:**
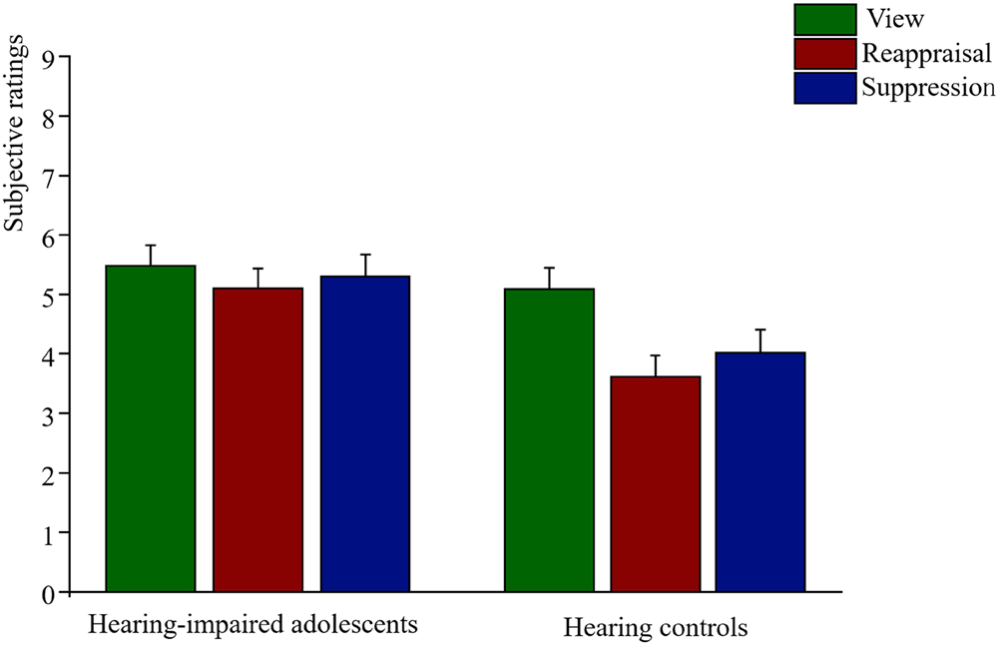
Negativity of social pain pictures in three conditions across two groups. Error bar indicates standard error (SE).

As presented in Table S2 (see Supplementary Materials) and Figure 2, the repeated‐measures ANOVA results showed a significant main effect of condition (*F*(2, 122) = 11.54, *p* < 0.001, *η* ^2^ = 0.159), such that the participants self‐reported less social negativity in the reappraisal condition and suppression condition than in the view condition. The main effect of the group was also found to be significant (*F*(1, 61) = 5.52, *p* < 0.05, *η* ^2^ = 0.083): hearing‐impaired adolescents versus hearing controls demonstrated more social negativity. More importantly, we found the significant group‐by‐condition interaction (*F*(2, 122) = 4.36, *p* < 0.05, *η* ^2^ = 0.067). The results of post‐hoc comparison showed that for hearing controls, compared with viewing, the use of reappraisal or suppression strategy effectively decreased negativity induced by social exclusion pictures. However, the use of emotion regulation strategies did not influence the negativity of social pain pictures in hearing‐impaired adolescents.

### 3.2 ERP Results

#### 3.2.1 P3

**FIGURE 3:**
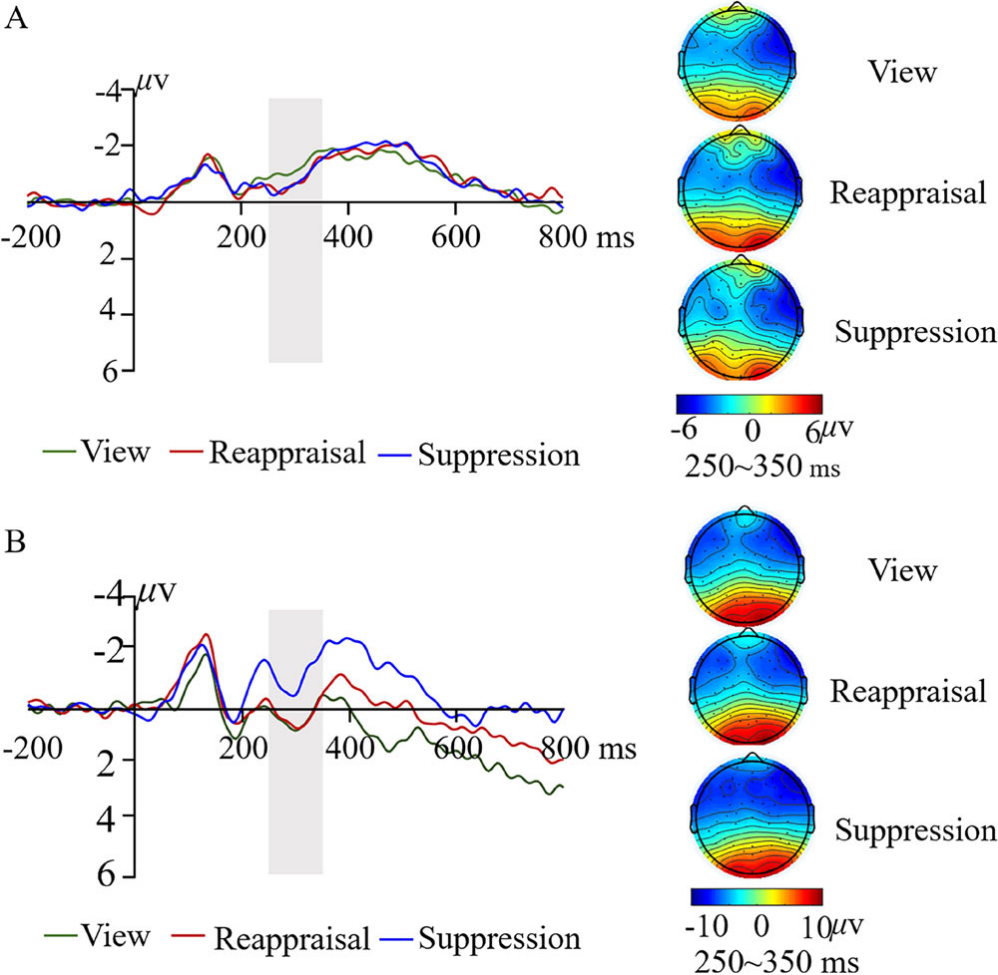
Waveforms and topographical maps of P3 at central‐parietal areas in hearing‐impaired adolescents (A) and hearing controls (B).

The results of the P3 component are summarized in Table S2. Figure 3 presents grand‐averaged waveforms and topographical maps in three conditions across two groups. The interaction between group and condition was found to be significant (*F*(2, 122) = 5.32, *p* < 0.01, *η* ^2^ = 0.080). The results of post‐hoc tests demonstrated that in hearing controls, the suppression strategy evoked smaller P3 amplitudes than the view and reappraisal strategies. However, no difference was found in three conditions in hearing‐impaired adolescents. The difference across conditions indicated a trend‐level significant effect (*F*(2, 122) = 2.48, *p* = 0.088), demonstrating that the reappraisal strategy induced more positive P3 as compared to the suppression strategy. The main effect of the group failed to reach significance (*F*(1, 61) = 0.68, *p* = 0.357).

#### 3.2.2 LPP

**FIGURE 4:**
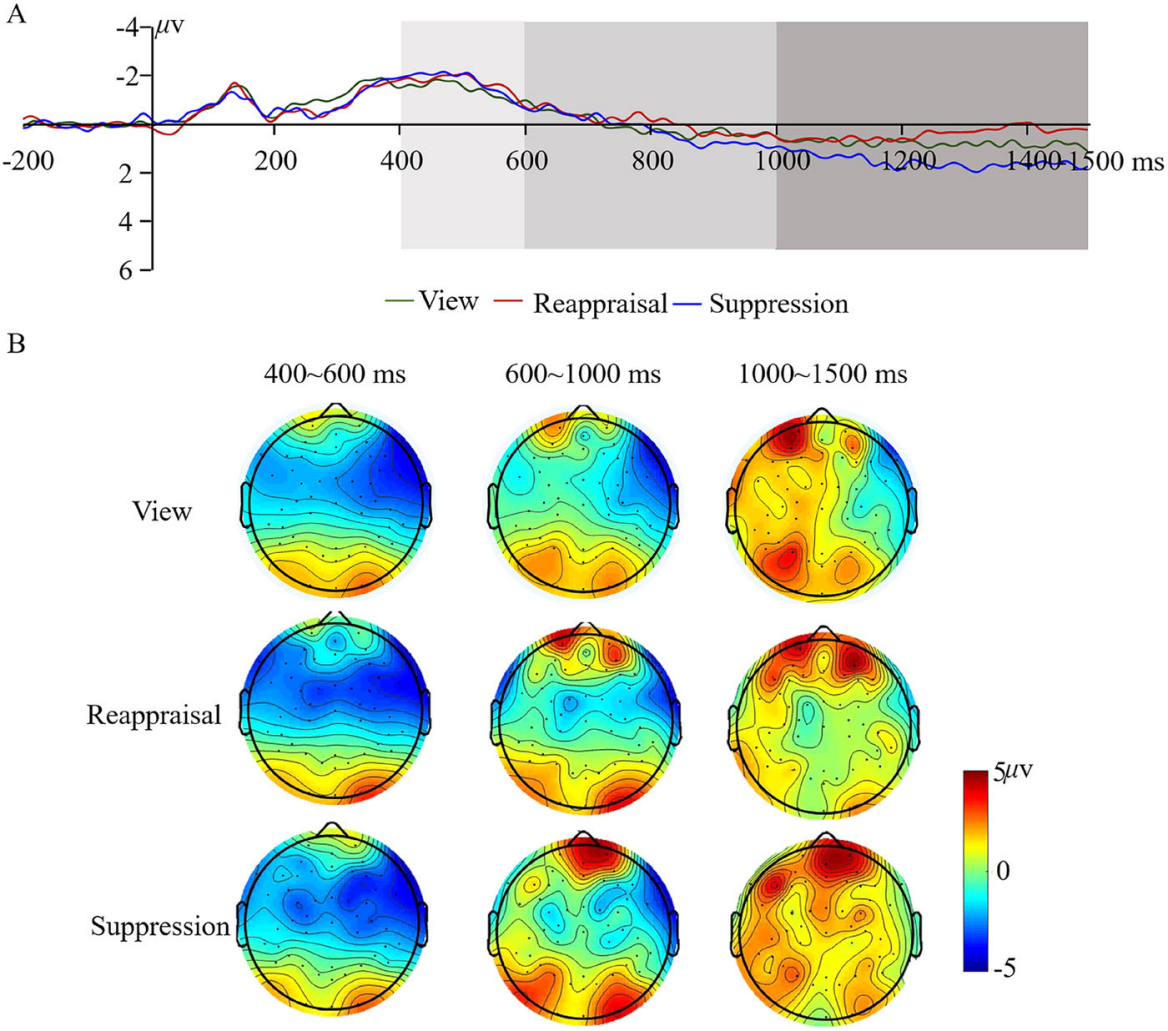
Waveforms (A) and topographical maps (B) of LPP at central‐parietal areas in hearing‐impaired adolescents.

**FIGURE 5:**
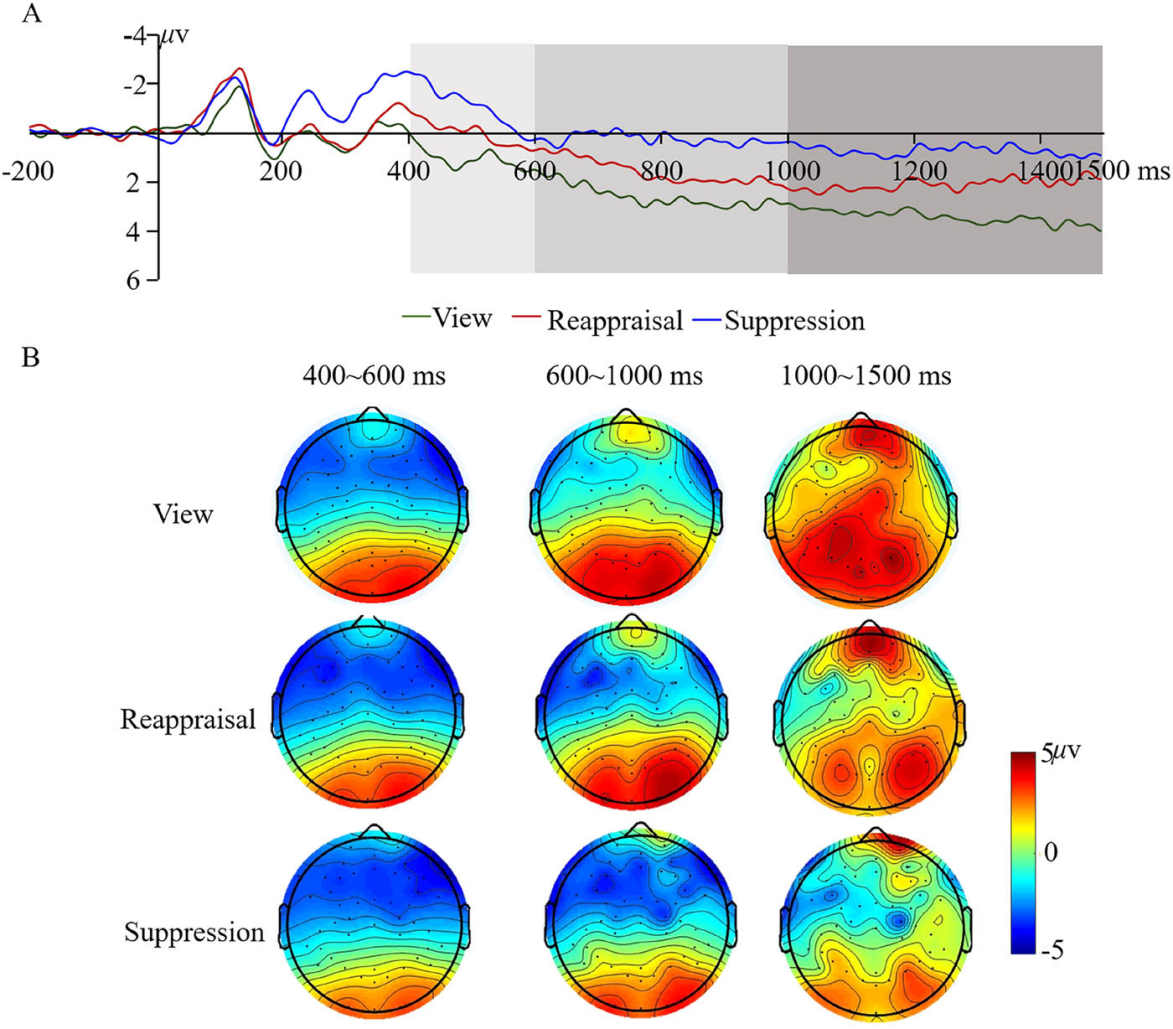
Waveforms (A) and topographical maps (B) of LPP at central‐parietal areas in hearing controls.

Building on previous literature (Desatnik et al. 2017; Yang et al. 2021), we examined the time course of different emotion regulation strategies by dividing the mean amplitudes of the LPP across the 400‐1500 ms time window into three consecutive segments. These segments included an early time window (400‐600 ms), a middle time window (600‐1000 ms), and a late time window (1000‐15000 ms). A repeated measures ANOVA with group as the between‐subjects factor and condition as the within‐subjects factor was performed separately in different time windows. Figures 4 and 5 present grand averaged ERP and topographical maps for hearing‐impaired adolescents and hearing controls, including view, reappraisal, and suppression conditions. Means and standard deviations (SD) were summarized in Table S2.

### 3.3 Early Time Window

The results of repeated‐measures ANOVA demonstrated a significant main effect of condition (*F*(2, 122) = 5.32, *p* < 0.01, *η* ^2^ = 0.080), whereby the main effect of group was found to be marginally significant (*F*(1, 61) = 2.80, *p* = 0.099). Interestingly, the interaction between group and condition was found to be significant (*F*(2, 122) = 3.15, *p* < 0.05, *η* ^2^ = 0.049). The results of the post‐hoc comparison demonstrated the suppression strategy had an obvious emotional regulation effect on social negativity in hearing controls, as indicated by the attenuated LPP for the suppression condition compared with the view condition. However, this emotional regulation effect did not reach significance in hearing‐impaired adolescents.

### 3.4 Middle Time Window

The main effect of the condition was found to be marginally significant (*F*(2, 122) = 2.39, *p* = 0.096). The main effect of the group was also marginally significant (*F*(1, 61) = 3.32, *p* = 0.074). More importantly, the interaction of group and condition was also marginally significant (*F*(2, 122) = 3.05, *p* = 0.051). The results of the post‐hoc comparison showed the effect of the suppression strategy on LPP was marginally significant only for hearing controls, as reflected by more positive amplitudes in the view condition than in the suppression condition.

### 3.5 Late Time Window

The interaction between group and condition was significant (*F*(2, 122) = 3.91, *p* < 0.05, *η* ^2^ = 0.060). The results of *post‐hoc* comparison demonstrated that compared with passively viewing social exclusion pictures, suppressing emotional response to social exclusion pictures attenuated amplitudes of LPP in hearing controls. However, there were no significant differences across the three conditions in hearing‐impaired adolescents. Neither the main effects of the group nor the condition were significant (Group: *F*(1, 61) = 2.31, *p* = 0.134; Condition: *F*(2, 122) = 1.41, *p* = 0.247).

## 4 Discussion

This study is the first to investigate the impact of hearing impairment on emotion regulation among adolescents in social contexts. Consistent with our hypotheses, behavioral results indicated that compared to passive viewing, both reappraisal and suppression significantly reduced negativity elicited by social exclusion scenarios in hearing controls, but not in hearing‐impaired adolescents. This suggests that hearing‐impaired adolescents may be unable to effectively employ reappraisal or suppression to down‐regulate negative social emotion.

Neurophysiological findings revealed that down‐regulating negative social emotion via expression suppression led to attenuated P3 amplitudes in hearing controls, whereas suppression did not modulate P3 responses in hearing‐impaired adolescents. Furthermore, in line with previous clinical studies (Bartolomeo et al. 2020; Hoid et al. 2020), hearing‐impaired adolescents did not exhibit the typical central‐parietal attenuation of the LPP under emotion regulation conditions. Specifically, hearing controls showed reduced early and late LPP amplitudes during suppression compared to viewing, while hearing‐impaired adolescents displayed comparable early and late LPP amplitudes across conditions. These ERP results indicate that hearing impairment may contribute to deficits in social‐emotional regulation during adolescence.

Previous research by Wang et al. (2016) suggests that hearing impairment may severely affect the maturation and development of the ventrolateral prefrontal cortex (VLPFC), a region crucial for down‐regulating negative social emotion (Li et al. 2022). Given that the central‐parietal P3 reflects attentional allocation to salient stimuli (Hajcak et al. 2010), and the LPP reflects subsequent elaborative processing (Dennis and Hajcak 2009; Keil et al. 2022; Usler and Weber 2021; Yan et al. 2022), our findings suggest that hearing impairment may hinder adolescents’ ability to reduce cognitive load during early‐stage social‐emotional processing by consciously allocating fewer attentional resources to emotional stimuli. This may compromise the efficient allocation of resources needed for deeper emotional information processing.

Interestingly, although reappraisal and suppression both effectively downregulated negative emotion triggered by social exclusion scenarios at the behavioral level in hearing controls, only suppression rather than reappraisal modulated ERP amplitudes at the neural level in this group, specifically those of the P3, early LPP, and late LPP. This suggests that suppression may be a more effective strategy than reappraisal for down‐regulating negative social emotion in this group. One possible explanation is that suppression requires fewer attentional resources than reappraisal during early emotional processing (Yan et al. 2022). Given limited attentional capacity, typically hearing adolescents may thus be more adept at using suppression to mitigate negative social emotions. Additionally, cultural context may help interpret these results. Conducted with Chinese adolescents, this study aligns with previous research indicating that suppression is often more effective than reappraisal in down‐regulating negative emotions within Asian cultural contexts (Lennarz et al. 2019; Yao and Xu 2023; Yuan et al. 2015). These consistent findings highlight the cultural relevance of emotion regulation strategies.

While this study yielded several meaningful findings, some limitations should be considered. Although this study specified that all hearing‐impaired adolescents were recruited from a public special school, it did not report further participant characteristics. Given the heterogeneity of this group, the absence of details regarding the severity of hearing loss, use of assistive devices, socioeconomic status, and educational background constitutes a limitation, as these factors are known to influence emotional functioning (Awan et al. 2024; Khalid et al. 2025). Beyond the limitation in participant characteristic reporting, the ecological validity of social‐emotional regulation was also not assessed in this study. Notably, this research focused exclusively on Chinese adolescents, a sample reflecting a collectivistic cultural context, and specifically examined the neural mechanisms behind cognitive reappraisal and expressive suppression when regulating negative social emotion. This aligns with findings from a systematic review: individualistic cultures tend to prioritize emotional expression as an emotion regulation strategy, whereas collectivistic cultures (like that of this study's Chinese participants) place greater emphasis on expressive suppression (Ramzan and Amjad 2017). These limitations, including incomplete measurement of key participant characteristics, unassessed ecological validity, and cultural specificity to collectivistic contexts, mean the generalizability of this study's findings requires careful consideration. Another limitation of this study is that it did not distinguish between the two subtypes of cognitive reappraisal: reinterpretation and distancing. These two tactics are known to involve distinct neural mechanisms during emotion regulation (Hermann et al. 2021; Qi et al. 2020; Rompilla et al. 2022). As such, future research should explicitly explore how these strategies differ in their effects on downregulating negative social emotion in adolescents with hearing impairment.

## 5 Conclusion

In conclusion, this study not only found impaired regulation of negative social emotions among hearing‐impaired adolescents but also further identified the specific processing stages at which these regulatory abnormalities occur. Beyond advancing our understanding of emotion regulation within social interaction contexts, these findings offer a fresh avenue for developing targeted interventions for emotional disorders in this population. This is a critical gap toward addressing the unique emotional challenges faced by hearing‐impaired adolescents, who often navigate social exchanges with distinct sensory and communicative constraints. For instance, existing research has consistently shown the ventrolateral prefrontal cortex (VLPFC) to play a pivotal role in downregulating negative social emotion (He et al. 2018; Li et al. 2022). Building on this, the current study's insights suggest this brain region could serve as a key clinical target for mitigating emotion dysregulation in hearing‐impaired adolescents with emotional disorders. By centering the VLPFC, clinicians might explore targeted approaches such as neurofeedback training to strengthen VLPFC activity or combined neuromodulation and behavioral therapy. These approaches can directly address the neural underpinnings of their emotion regulation struggles rather than just managing surface‐level symptoms.

## Author Contributions

**Panpan Zhang**: material preparation, data collection, formal analysis, writing – original draft. **Minghui Wang**: writing – original draft, writing – review and editing. **Huang Gu**: conceptualization, supervision, writing – review and editing.

## Funding

This work was supported by the Humanities and Social Science Fund of the Ministry of Education of China [grant number 23YJCZH056].

## Ethics Statement

All procedures performed in studies involving human participants were by the ethical standards of the institutional and/or national research committee and with the 1964 Helsinki Declaration and its later amendments or comparable ethical standards. The study was approved by the Institutional Review Board of Henan Provincial Key Laboratory of Psychology and Behavior (No. 20230208001).

## Consent

Informed consent was obtained from the parents.

## Conflicts of Interest

The authors declare no conflicts of interest.

## Supporting information




**Supplementary Material**: brb371080‐sup‐0001‐tableS1‐S2.docx

## Data Availability

Derived data supporting the findings of this study are available from the corresponding author Huang Gu on request.

## References

[brb371080-bib-0001] Awan, N. U. , U. Malik , M. Azam , et al. 2024. “Behavioral and Emotional Difficulties in Hearing impaired Children and Adolescents: A Systematic Review.” Kurdish Studies 12, no. 4: 544–554.

[brb371080-bib-0002] Bartolomeo, L. A. , A. J. Culbreth , K. L. Ossenfort , and G. P. Strauss . 2020. “Neurophysiological Evidence for Emotion Regulation Impairment in Schizophrenia: The Role of Visual Attention and Cognitive Effort.” Journal of Abnormal Psychology 129, no. 6: 670–676. 32525326 10.1037/abn0000580 PMC7440778

[brb371080-bib-0003] Cejas, I. , J. Coto , C. Sanchez , M. Holcomb , and N. E. Lorenzo . 2021. “Prevalence of Depression and Anxiety in Adolescents With Hearing Loss.” Otology & Neurotology 42, no. 4: e470–e475. 33347049 10.1097/MAO.0000000000003006

[brb371080-bib-0004] Cupples, L. , T. Y. Ching , L. Button , et al. 2018. “Language and Speech Outcomes of Children With Hearing Loss and Additional Disabilities: Identifying The Variables That Influence Performance at Five Years of Age.” International Journal of Audiology 57, no. S2: S93–S104. 27630013 10.1080/14992027.2016.1228127 PMC5350072

[brb371080-bib-0005] Dennis, T. A. , and G. Hajcak . 2009. “The Late Positive Potential: A Neurophysiological Marker for Emotion Regulation in Children.” Journal of Child Psychology and Psychiatry 50, no. 11: 1373–1383. 19754501 10.1111/j.1469-7610.2009.02168.x PMC3019134

[brb371080-bib-0006] Desatnik, A. , T. Bel‐Bahar , T. Nolte , M. Crowley , P. Fonagy , and P. Fearon . 2017. “Emotion Regulation in Adolescents: An ERP Study.” Biological Psychology 129: 52–61. 28803782 10.1016/j.biopsycho.2017.08.001

[brb371080-bib-0007] Eisenberger, N. I. 2012. “The Neural Bases of Social Pain: Evidence for Shared Representations With Physical Pain.” Psychosomatic Medicine 74, no. 2: 126–135. 22286852 10.1097/PSY.0b013e3182464dd1 PMC3273616

[brb371080-bib-0008] Fitzpatrick, E. M. , I. Gaboury , A. Durieux‐Smith , D. Coyle , J. Whittingham , and F. Nassrallah . 2019. “Auditory and Language Outcomes in Children With Unilateral Hearing Loss.” Hearing Research 372: 42–51. 29573881 10.1016/j.heares.2018.03.015

[brb371080-bib-0009] Gross, J. J. 1998a. “Antecedent‐ and Response‐Focused Emotion Regulation: Divergent Consequences for Experience, Expression, and Physiology.” Journal of Personality and Social Psychology 74, no. 1: 224–237. 9457784 10.1037//0022-3514.74.1.224

[brb371080-bib-0010] Gross, J. J. 1998b. “The Emerging Field of Emotion Regulation: An Integrative Review.” Review of General Psychology 2, no. 3: 271–299.

[brb371080-bib-0011] Hajcak, G. , A. MacNamara , and D. M. Olvet . 2010. “Event‐Related Potentials, Emotion, and Emotion Regulation: An Integrative Review.” Developmental Neuropsychology 35, no. 2: 129–155. 20390599 10.1080/87565640903526504

[brb371080-bib-0012] He, Z. , Y. Lin , L. Xia , Z. Liu , D. Zhang , and R. Elliott . 2018. “Critical Role of the Right VLPFC in Emotional Regulation of Social Exclusion: A tDCS Study.” Social Cognitive and Affective Neuroscience 13, no. 4: 357–366. 29618116 10.1093/scan/nsy026 PMC5928413

[brb371080-bib-0014] He, Z. , N. Muhlert , and R. Elliott . 2021. “Emotion Regulation of Social Exclusion: A Cross‐Cultural Study.” Humanities and Social Sciences Communications 8, no. 1: 1–7.38617731

[brb371080-bib-0015] He, Z. , J. Zhao , J. Shen , N. Muhlert , R. Elliott , and D. Zhang . 2020. “The Right VLPFC and Downregulation of Social Pain: A TMS Study.” Human Brain Mapping 41, no. 5: 1362–1371. 31789480 10.1002/hbm.24881 PMC7267938

[brb371080-bib-0016] Hermann, A. , M. K. Neudert , A. Schäfer , et al. 2021. “Lasting Effects of Cognitive Emotion Regulation: Neural Correlates of Reinterpretation and Distancing.” Social Cognitive and Affective Neuroscience 16, no. 3: 268–279. 33227135 10.1093/scan/nsaa159 PMC7943369

[brb371080-bib-0017] Hoid, D. , D.‐N. Pan , Y. Wang , and X. Li . 2020. “Implicit Emotion Regulation Deficits in Individuals With High Schizotypal Traits: An ERP Study.” Scientific Reports 10, no. 1: 3882. 32127580 10.1038/s41598-020-60787-9 PMC7054415

[brb371080-bib-0018] Jauch, M. , S. C. Rudert , and R. Greifeneder . 2022. “Social Pain by Non‐Social Agents: Exclusion Hurts and Provokes Punishment Even if the Excluding Source is a Computer.” Acta Psychologica 230: 103753. 36166852 10.1016/j.actpsy.2022.103753

[brb371080-bib-0019] Keil, V. , B. Tuschen‐Caffier , and J. Schmitz . 2022. “Effects of Cognitive Reappraisal on Subjective and Neural Reactivity to Angry Faces in Children With Social Anxiety Disorder, Clinical Controls With Mixed Anxiety Disorders and Healthy Children.” Child Psychiatry and Human Development 53, no. 5: 886–898. 33895894 10.1007/s10578-021-01173-y PMC9470612

[brb371080-bib-0020] Khalid, U. , N. Majeed , C. J. Chovaz , F. R. Choudhary , and K. Munawa . 2025. “Psychological Well‐Being and Mental Health Risks in Deaf and Hard of Hearing Youth: A Systematic Review.” European Child & Adolescent Psychiatry Advance online publication. 10.1007/s00787-025-02795-6 40569433

[brb371080-bib-0021] Lennarz, H. K. , T. Hollenstein , A. Lichtwarck‐Aschoff , E. Kuntsche , and I. Granic . 2019. “Emotion Regulation in Action: Use, Selection, and Success of Emotion Regulation in Adolescents' Daily Lives.” International Journal of Behavioral Development 43, no. 1: 1–11. 30613118 10.1177/0165025418755540 PMC6305959

[brb371080-bib-0022] Li, S. , H. Xie , Z. Zheng , et al. 2022. “The Causal Role of The Bilateral Ventrolateral Prefrontal Cortices on emotion Regulation of Social Feedback.” Human Brain Mapping 43, no. 9: 2898–2910. 35261115 10.1002/hbm.25824 PMC9120569

[brb371080-bib-0023] Olsson, S. , M. Dag , and C. Kullberg . 2021. “Hard of Hearing Adults' Interpersonal Interactions and Relationships in Daily Life.” Disabilities 1, no. 2: 71–88.

[brb371080-bib-0024] Qi, S. , J. Basanovic , L. Wang , S. Xiang , W. Hu , and X. Yi . 2020. “Regulation of Negative Emotions Through Positive Reappraisal and Distancing in High‐Trait‐Anxious Women.” Journal of Affective Disorders 267: 191–202. 32217219 10.1016/j.jad.2020.02.027

[brb371080-bib-0025] Ramzan, N. , and N. Amjad . 2017. “Cross Cultural Variation in Emotion Regulation: A Systematic Review.” Annals of King Edward Medical University 23, no. 1: 77–90.

[brb371080-bib-0026] Rapee, R. M. , E. L. Oar , C. J. Johnco , et al. 2019. “Adolescent Development and Risk for the Onset of Social‐Emotional Disorders: A Review and Conceptual Model.” Behaviour Research and Therapy 123: 103501. 31733812 10.1016/j.brat.2019.103501

[brb371080-bib-0027] Reinhard, M. A. , J. Dewald‐Kaufmann , T. Wuestenberg , et al. 2020. “The Vicious Circle of Social Exclusion and Psychopathology: A Systematic Review of Experimental Ostracism Research in Psychiatric Disorders.” European Archives of Psychiatry and Clinical Neuroscience 270, no. 5: 521–532. 31586242 10.1007/s00406-019-01074-1

[brb371080-bib-0028] Rompilla Jr, D. B. , E. F. Hittner , J. E. Stephens , I. Mauss , and C. M. Haase . 2022. “Emotion Regulation in the Face of Loss: How Detachment, Positive Reappraisal, and Acceptance Shape Experiences, Physiology, and Perceptions in Late Life.” Emotion (Washington, DC) 22, no. 7: 1417–1434. 10.1037/emo0000932 33661660

[brb371080-bib-0029] Socher, M. , B. Lyxell , R. Ellis , M. Gärskog , I. Hedström , and M. Wass . 2019. “Pragmatic Language Skills: A Comparison of Children With Cochlear Implants and Children Without Hearing Loss.” Frontiers in Psychology 10: 2243. 31649586 10.3389/fpsyg.2019.02243 PMC6794448

[brb371080-bib-0030] Sun, Y. , J. Lv , F. Lan , and L. Zhang . 2020. “Emotion Regulation Strategy of Self‐Focused and Situation‐Focused Reappraisal and Their Impact on Subsequent Cognitive Control.” Acta Psychologica Sinica 52, no. 12: 1393–1406.

[brb371080-bib-0031] Usler, E. R. , and C. Weber . 2021. “Emotion Processing in Children Who do and do Not Stutter: An ERP Study of Electrocortical Reactivity and Regulation to Peer Facial Expressions.” Journal of Fluency Disorders 67: 105802. 33227619 10.1016/j.jfludis.2020.105802

[brb371080-bib-0032] Walker, E. A. , C. Sapp , M. Dallapiazza , M. Spratford , R. W. McCreery , and J. J. Oleson . 2020. “Language and Reading Outcomes in Fourth‐Grade Children With Mild Hearing Loss Compared to Age‐Matched Hearing Peers.” Language, Speech, and Hearing Services in Schools 51, no. 1: 17–28. 31913806 10.1044/2019_LSHSS-OCHL-19-0015 PMC7251588

[brb371080-bib-0033] Wang, X. , P. Xu , P. Li , et al. 2016. “Alterations in Gray Matter Volume Due to Unilateral Hearing Loss.” Scientific Reports 6: 25811. 27174521 10.1038/srep25811 PMC4865827

[brb371080-bib-0034] Yan, C. , Q. Ding , Y. Wang , M. Wu , T. Gao , and X. Liu . 2022. “The Effect of Cognitive Reappraisal and Expression Suppression on Sadness and the Recognition of Sad Scenes: An Event‐Related Potential Study.” Frontiers in Psychology 13: 935007. 36211892 10.3389/fpsyg.2022.935007 PMC9537681

[brb371080-bib-0035] Yang, M. , X. Deng , and S. An . 2021. “The Immediate and Lasting Effect of Emotion Regulation in Adolescents: An ERP Study.” International Journal of Environmental Research and Public Health 18, no. 19: 10242. 34639542 10.3390/ijerph181910242 PMC8549699

[brb371080-bib-0036] Yao, Y. , and D. Xu . 2023. “Unconscious Cognitive Reappraisal and Unconscious Expression Suppression Regulate Emotional Responses: An ERP Study.” Current Psychology 43: 7772–7784.

[brb371080-bib-0037] Yuan, J. , Q. Long , N. Ding , Y. Lou , Y. Liu , and J. Yang . 2015. “Suppression Dampens Unpleasant Emotion Faster Than Reappraisal: Neural Dynamics in a Chinese Sample.” Science China Life Sciences 58, no. 5: 480–491. 25316046 10.1007/s11427-014-4739-6

[brb371080-bib-0038] Zhao, J. , L. Mo , R. Bi , et al. 2021. “The VLPFC versus the DLPFC In Downregulating Social Pain Using Reappraisal and Distraction Strategies.” Journal of Neuroscience 41, no. 6: 1331–1339. 33443069 10.1523/JNEUROSCI.1906-20.2020 PMC7888223

[brb371080-bib-0039] Zheng, Z. , S. Li , L. Mo , W. Chen , and D. Zhang . 2022. “ISIEA: An Image Database of Social Inclusion and Exclusion in Young Asian Adults.” Behavior Research Methods 54, no. 5: 2409–2421. 34918228 10.3758/s13428-021-01736-w PMC9579065

