# Supplementary material for: Socially‐Based Emotion Dysregulation Among Hearing‐Impaired Adolescents: An Event‐Related Potential Study
Source: Brain Behav. 2025 Nov 21;15(11):e71080. doi: 10.1002/brb3.71080 (PMC12638441; doi:10.1002/brb3.71080)
Supplement: Supplementary file 1 — Supplementary Material: brb371080‐sup‐0001‐tableS1‐S2.docx [file BRB3-15-e71080-s001.docx]

**Table S1**. The number of remaining trails in three conditions across groups.

|  | View | Reappraisal | Suppression | *F_Group_* | *F_Condition_* | *F_Group*Condition_* |
| --- | --- | --- | --- | --- | --- | --- |
| Hearing-impaired adolescents | 28.15±2.24 | 28.42±2.33 | 28.33±2.34 | 0.23 | 1.12 | 0.12 |
| Hearing controls | 27.73±3.10 | 28.47±2.49 | 28.20±2.62 |  |  |  |

**Table S2**. Descriptive statistics of ratings and amplitudes of ERP components between hearing-impaired adolescents and hearing controls.

|  | Hearing-impaired adolescents | | |  | Hearing controls | | | *F*_Group_ | *F*_Contion_ | *F*_Group*Contion_ |
| --- | --- | --- | --- | --- | --- | --- | --- | --- | --- | --- |
|  | View | Reappraisal | Suppression |  | View | Reappraisal | Suppression |  |  |  |
| Ratings | 5.48 ± 1.90 | 5.10 ± 1.79 | 5.30 ± 1.98 |  | 5.09 ± 1.96 | 3.62 ± 2.05 | 4.02 ± 2.29 | 5.52^*^ | 11.54^*^^**^ | 4.36^*^ |
| P3 | -1.21 ± 3.52 | -0.76 ± 2.99 | -0.72 ± 3.42 |  | 0.34 ± 3.11 | 0.27 ± 3.95 | -1.10 ± 3.60 | 0.68 | 2.48 | 5.32^**^ |
| LPP |  |  |  |  |  |  |  |  |  |  |
| 400~600 | -1.43 ± 3.39 | -1.71 ± 3.96 | -1.72 ± 4.40 |  | 1.07 ± 3.97 | 0.02 ± 5.05 | -1.11 ± 4.26 | 2.80 | 5.32^**^ | 3.15^*^ |
| 600~1000 | 0.08 ± 3.20 | -0.08 ± 4.49 | 0.21 ± 4.33 |  | 2.58 ± 2.80 | 1.58 ± 4.12 | 0.26 ± 3.68 | 3.32 | 2.39 | 3.05 |
| 1000~1500 | 0.80 ± 3.03 | 0.40 ± 5.69 | 1.54 ± 4.60 |  | 3.43 ± 3.10 | 2.10 ± 4.08 | 0.73 ± 4.17 | 2.31 | 1.41 | 3.91^*^ |

^*^ *p* < 0.05, ^**^ *p* < 0.01, ^***^ *p* < 0.001.
